# Sun-health behaviours and attitudes towards sun safety amongst Australian teenagers: a qualitative update

**DOI:** 10.1186/s13104-021-05764-9

**Published:** 2021-09-08

**Authors:** Nisali Gamage, Rebecca Nguyen, Isabelle M. Clare, Robyn M. Lucas, Mark Strickland, Joanna Granich, Shelley Gorman

**Affiliations:** 1 Telethon Kids Institute, University of Western Australia, 15 Hospital Ave, Nedlands, WA 6008 Australia; 2 National Centre for Epidemiology and Population Health, Research School of Population Health, Australian National University, Canberra, ACT Australia; 3 Centre for Ophthalmology and Visual Science, University of Western Australia, Perth, Australia; 4 Cancer Council Western Australia, Perth, Australia

**Keywords:** Attitudes, Behaviours, Knowledge gaps, Personal considerations, Sun exposure, Sun protection, UV Index, Vitamin D, Young adolescents

## Abstract

**Objective:**

This study aimed to explore current attitudes towards sun protection, and sun-seeking behaviour among young Australian adolescents. It was done as part of a larger project aiming to develop a digital resource to support young people in making informed sun-health decisions.

**Results:**

Ten (4 male, 6 female) adolescents (12–13 years of age) living in Perth (Western Australia) were recruited through a social media-based strategy. Each participant engaged in a semi-structured telephone interview which explored their sun-health decision-making, with interview transcripts assessed qualitatively using NVivo. Three major themes (and eight sub-themes) were identified: (1) ‘personal sun health considerations’; (2) ‘attitudes towards sun protection’; and (3) ‘recommendations’. The importance of sun protection was appreciated by participants. However, females were more diligent in the use of sun protection while males were indifferent. Behaviours were influenced by parental input, the school environment and engagement in sport. Adolescents had limited knowledge of the UV Index and its implications for sun protection, and the health importance of sun-derived vitamin D. Overall, the importance of sun protection was acknowledged but did not consistently translate into sun protective behaviours.

## Introduction

Data for this study comes from a larger project, in which we developed a new digital resource—the *Sun Safe* iOS smartphone app [1, 2]. This resource was developed to assist young people in making healthier decisions around spending time in the sun. Skin cancer is the most commonly diagnosed cancer in Australia. Repeated episodes of severe sunburn during childhood and adolescence may confer particular risk for melanoma, the most lethal skin cancer [3, 4]. Melanoma is the most commonly diagnosed cancer amongst young people (aged 15–24 years) in Australia, accounting for 15% of cancer diagnoses in this age group [5]. Despite the risks of sun exposure, some sun exposure is required for vitamin D synthesis. Unexpectedly, vitamin D deficiency was detected in ~ 29% of young adults (25–34 year-olds) in the most recent Australian Health Survey (2011–13) [6, 7]. New resources are thus needed to establish life-long behaviours by young people to mitigate risks for skin cancer while not compromising sun exposure for vitamin D.

The co-design methodology underpinning development of the *Sun Safe* app is described in detail elsewhere [2]. These methods embraced a user-centred Design Thinking process based on the 5 stage Stanford process (empathise, define, ideate, prototype, test) [2, 8, 9]. Telephone interviews of 10 young people (12–13 year-olds) were done during the empathise and define stages of the research process and allowed researchers to understand end-users’ perspectives regarding their sun-health with insights gained informing the development of the *Sun Safe* app [2]. Data described here are qualitative analyses done of transcripts from these telephone interviews.

## Main text

### Ethics

Approval to conduct this study was obtained from the Human Research Ethics Committee of the University of Western Australia (RA/4/20/4424), which was conducted according to COnsolidated criteria for REporting Qualitative research (COREQ) guidelines.

### Participant recruitment

For more details on the study location, and recruitment methods, see reference [2]. We recruited young adolescents through a social media strategy in August 2018. Inclusion criteria were that participants: were 12–13 years of age; resided in Perth (WA); had access to the internet and an iOS device (such as iPhone or iPad); and, could understand and speak English. Informed written consent to conduct the recorded interview was obtained from adolescents and their parent/guardian.

### Semi-structured interviews

Individual participants were interviewed by telephone with responses elicited using a semi-structured framework (Table 1). Interviews were conducted by author RN who worked as a Digital Health Research Officer on this project, utilising her 3 years of experience as a Youth Consumer Advocate [with the Consumer and Community Health Research Network (Telethon Kids Institute), the Commissioner for Children and Youth People, and Department of Health (Western Australia)] and expertise gained conducting interviews with young people that also have used Design Thinking frameworks (e.g., for the Cystic Fibrosis app [10]). RN has relevant qualifications from Stanford University (General Assembly, Design Thinking course), Echos—Innovations Lab (Design Thinking course) and Curtin University [Bachelor of Commerce (Information Technology)]. At the beginning of each interview, the interviewer introduced herself as a researcher, outlined the purpose of the study, and encouraged candid responses, reassuring the participant that there were no right or wrong answers as researchers were interested in hearing participants’ experiences and recommendations. Participants were then asked a series of questions (Table 1) with further questions to elaborate pertinent points. No repeat interviews were conducted. Further details can be read in reference [2].

**Table 1:** Interview question framework

Questions
1. Thinking of the last time you went out on a hot day this summer, where did you go, and what did you do?
2. How did the weather affect you, in terms of getting ready?
3. Do you think about these things when you get ready for school during summer?
4. What about getting ready for school during winter?
5. What about on the way home from school or on the weekend?
6. Do you check the weather, and if so, how?
7. How do you feel about sun protection? Is it a concern to you?
8. Is there anything we haven’t asked you about your sun-health and how you use technology and that you would like to tell us that we should consider?

### Analysis of interviews

Descriptive data analyses (mean, range and SD) were conducted in Prism GraphPad (v8.4.3, 2020). Interviews were transcribed verbatim and cross-checked against recorded audio for accuracy. NG de-identified transcripts and allocated each a unique identification number. Transcripts were then imported into NVivo 12 (2019). Formal thematic data analyses, underpinned by a phenomenological framework [11, 12], were done by NG. Common and novel themes were identified and categorised, fitting with questions and topics covered in the interviews [13]. Two independent researchers (SG, RN) previously informally and rapidly coded data from interviews into themes, which were explored further in workshops done with co-researchers for development of the *Sun Safe* iOS app (described in detail in [2]). Findings from these independent theming (i.e. formal and informal) analyses are compared below in “Limitations” section.

### Participant demographics

Ten (10) participants were recruited, including 4 males and 6 females, with a mean age of 12.8 ± 0.4 years (mean ± SD). The mean interview length for all participants was 32:52 min:s (range = 24:51–44:50 min:s). The interview lengths for male (mean = 30:34 min:s) and female (mean length = 34:25 min:s) participants were similar.

### Qualitative findings—overview

Interviews provided insight into adolescents’ daily routines and attitudes towards sun protection. Overall, most adolescents were sun-seeking, enjoyed spending time outdoors and recognised the importance of sun protection. However, a sense of indifference was common among male participants. The analyses also identified participants’ knowledge gaps and recommendations towards developing an online tool to inform sun-health decision making. The major themes and sub-themes identified are summarised in Table 2.

**Table 2:** Major themes and sub-themes identified from semi-structured interviews of young adolescents (12–13 years-old) that explored their experiences, and attitudes and behaviours towards sun protection

Themes	Sub-themes
1. Personal sun health considerations	1.1 Typical activities included: going to the beach (n = 5; 4 female, 1 male); parks (n = 3)^a^; pool parties (n = 1); playing with pets (n = 1) 1.2 Checking the weather (using weather application on smartphone, asking parents, television weather report) (n = 7; 6 female, 1 male) 1.3 Sun protective behaviours included: applying sunscreen (n = 10); seeking shade (n = 5)
2. Attitudes towards sun protection	2.1 General attitudes (females were motivated (n = 6 of 6), males were indifferent (n = 3 of 4)) 2.2 Hats (recognised as a means of sun protection (n = 9); not trendy (n = 3)) 2.3 Influences (school environment (n = 7), community and school sports (n = 3), parents (n = 8))
3. Recommendations	3.1 Knowledge gaps (UV Index and implications for sun protection (n = 6); sun protection factor (SPF) (n = 2); benefits of sun exposure, including vitamin D (n = 1)) 3.2 Online tool input (consequences of excessive sun exposure, including anecdotal stories (n = 6); information regarding the UV Index and appropriate use of sunscreen (n = 6); mini-games or quizzes to improve knowledge (n = 2))

^a^n = number of participants who provided data for each sub-theme

### Theme 1: personal sun health considerations

#### Sub-theme 1.1: typical activities

Adolescents reported activities involving time spent outdoors in the sun on hot days. Pool parties, going to the park, beach and visiting adventure/theme parks were cited. A sense of enjoyment of time spent outdoors on warm sunny days and engagement in leisure activities was reported by most (9 of 10) adolescents (Table 3).

**Table 3:** Quotes from young adolescent participants conveying major sub-themes identified from interviews

Sub-themes	Participant (P) quotes
Theme 1—personal sun health considerations	
1.1: Typical activities	“Leisure swimming, I like … I like going to the beach with my friends.” (P6, female) “I was probably out back … a big, grassed area … playing with our pets.” (P10, female)
1.2: Influence of weather	“Say it’s going to be 30 [°C] or something that day and I know that we’re doing lots of sport that day, then I will bring sunscreen in my bag and apply a bit on my face.” (P3, female) “I usually use it [website with information on the predicted UV Index] when I have a sporting event on, or I have like PE in a later time of the day when I know I could get sunburnt.” (P8, female)
1.3: Sun protective behaviours	“We always make sure to put sunscreen on. Like it’s a habit.” (P6, female) “It makes me feel more secure that I’m not going to get sunburnt. I’ve put sunscreen on … it’s a very low chance that I do get sunburnt if I do have sunscreen on.” (P8, female) “Some days when I’m not sure whether it’s hot … I wait for Mum to tell me.” (P1, male)
Theme 2—attitudes towards sun protection	
2.1: General attitudes	“Well, I started packing um my sunscreen because I got really badly burnt one time and it was really painful.” (P3, female) “Like they might say ‘I only ever get burnt like once a year’… but that still…triples your chance for melanoma disease. People do need to be informed in that kind of way.” (P8, female) “It’s not so compulsory for me. Like it’s like I’m not scared of it or anything, but I know that it’s good for me…the reason I’m not scared of it is because nothing bad has happened to me yet…but I think if I had, I would be way more protective of myself” (P9, male)
2.2: Hats	“I mean if you’re going out in public, some hats would not be okay to wear if they are sort of not really like modern type.” (P1, male) “Yeah, girls don’t really wear hats because of their hair. Being a normal teenager worrying about that…” (P10, female) “I think it’s a good [sic] because it like stops your chances of getting burnt.” (P3, female)
2.3: Influences	“I don’t know if I’d want to wear a hat… like there’s no one else doing it, so…it would be different. But like I think if they made a rule to make it that you had to wear a hat, then like I’d have no problem wearing a hat.” (P6, female) “The PE staff always have sunscreen and like as people go out, everyone puts sunscreen on.” (P6, female) “Mum always says you need to put on sunscreen, otherwise you’ll have bad skin and skin problems when you’re older” (P6, female)
Theme 3—recommendations	
3.1: Knowledge gaps	“I don’t know too much about it, but I do know that too much UV can be harmful for you and that different sunscreens have different UV ratings and how much they’ll protect you.” (P5, female) “I don’t really know much about it. I just know that like things with higher SPF is generally what you want to go for.” (P6, female)
3.2: Online tool input	“Maybe some stories about people that have had too much exposure and what has happened to them. So that other people can get an idea that it’s better that you protect yourself from the sun.” (P5, female) “How much UV rays you would get out in the sun … the maximum amount of time you should be out in the sun without sunscreen.” (P5, female) “How much time people should spend in the sun each day so they get some vitamin D … and what could happen if you don’t have enough vitamin D.” (P5, female)

PE refers to school-based physical education classes

*SPF* sun protection factor

#### Sub-theme 1.2: checking the weather

Most adolescents independently checked the weather using their smartphone or device, via a weather app or website, or when watching the news on television (Table 3). Participants noted the maximum (predicted) temperature, cloud coverage or rainfall to guide their decision-making on sun protection. Some simply stepped outside to check for clouds, rain or sun. Often the weather was checked prior to outdoor physical activities. Only one interviewee mentioned the UV Index. Some participants were reliant and informed by their parents about the weather forecast (Table 3). Gender differences were observed with all female participants (*n* = 6) checking the weather frequently and concerned about what to wear. This appeared to be influenced by possible (adverse) health impacts that compelled them to use sun protection (Table 3)*.* Male participants seemed indifferent. Most (3 of 4) stated they did not regularly check the weather.

#### Sub-theme 1.3: sun protective behaviours

All participants cited sunscreen as the most commonly used method of sun protection (Table 3) as observed previously [14], with one participant describing it as ‘armour’ which protects the skin. However, gender differences with contrasting levels of motivation and concerns around sun protective behaviours were reported (Table 3). Female participants were more careful and adopted various lifestyle measures to ensure sun protection. They were also more diligent in their application of sunscreen (Table 3). Sun protection was acknowledged by males as important but did not appear to be such a priority as for females. During school recess and lunch breaks, male participants seemed to be more fun seeking, opting to play outside even if very hot and sunny, and were less proactive about wearing sunscreen. Males deemed sunscreen as annoying to apply and were unsure whether to apply it on most days or in mild weather.

### Theme 2: attitudes towards sun protection

#### Sub-theme 2.1: general attitudes

Overall, participants had positive attitudes towards sun protection, recognising that excessive sun exposure is damaging to skin. Previous sunburn (and pain experienced) was a compelling factor in the adoption of sun-safe behaviours (Table 3) as observed previously [15]. All participants had experienced sunburn, commonly citing that they forget to reapply sunscreen. School seminars and ‘testimonial’ advertisements on television featuring skin cancer patients appeared to positively influence adolescents’ sun protective attitudes. Again, females were more diligent about sun protection, while males seemed indifferent (Table 3).

#### Sub-theme 2.2: hats

Hats were identified as an important sun protective measure, but not a fashionable option (Table 3). Some occasionally reported wearing a hat opted for a cap, and not a more protective broad-brimmed hat.

#### Sub-theme 2.3: influences

Most adolescents reported that the school environment and their parents influenced their sun-health decisions (Table 3). However, several participants stated that most public high schools in Australia do not enforce a hat policy, with most participants and their peers not wearing hats when outdoors at school. Some participants attended private schools with mandatory hat-wearing policies. Whether hats should be enforced by schools for all year groups was an idea contemplated by one participant, based on concerns of adolescents about standing out from their peers (Table 3). Sports coaches were another crucial influence as they encouraged the *SunSmart* messages advocated by Australian Cancer Councils [16].

### Theme 3: recommendations

#### Sub-theme 3.1: knowledge gaps

Many participants (5 of 10) were unaware about the UV Index or were uncertain about what it meant (Table 3). One participant reported seeking to learn about the UV Index and how it impacted her risk of sunburn when playing netball outdoors. The sun protection factor (SPF) was another concept of uncertainty for participants (Table 3). There was limited discussion of sun exposure for vitamin D or other benefits.

#### Sub-theme 3.2: online tool input

Participants recommended that any developed online tool needed to be interactive, engaging and include stories and elements of mini-gaming or quizzes with simple facts and notifications as reminders (Table 3). Female participants expressed the need to learn more about the effectiveness of different types of sunscreens*,* UV Index ratings and vitamin D (Table 3). Other areas of interest included: learning about adverse impacts of UV radiation reflected off water; differing (skin) sensitivities to sun exposure; and methods of sun protection besides using sunscreen. One individual conveyed a novel idea about how climate change may impact sun protection.

## Limitations

Important limitations of this study were the small sample size (*n* = 10) and that all participants lived in metropolitan areas of Perth (Western Australia). This may limit the generalisability of findings to the wider Australian population and internationally. Another limitation relates to the social desirability response bias which is inherent in qualitative studies and may potentially skew responses around self-reported sun-related behaviours [17]. Many of the sub-themes identified across the themes of ‘personal sun health considerations’ and ‘attitudes towards sun protection’ were consistently observed across multiple participants. Unique and novel ideas were conveyed for development of the online tool, suggesting further interviews may be necessary to validate these ideas or achieve data saturation. Indeed, this was further explored as part of the Design Thinking process [8, 9] for the development of the *Sun Safe* app in which three additional workshops with participants (and other ‘co-researchers’) were conducted [2].

Reassuringly, themes that emerged from this qualitative analysis were similar to those previously identified during the more rapid Design Thinking process [2]. The themes identified in that previous analysis, included: (1) major strategies used by participants to make decisions (e.g. the weather forecast); (2) challenges (a lack of understanding of the UV Index); (3) emotions (e.g. anxiety around the consequences of excessive sun exposure); and, (4) needs (e.g. an informative online tool for young teenagers) [2]. Several recommendations from the interviews were translated into the development of the *Sun Safe* app, such as inclusion of the story of a young survivor of melanoma. The emotive nature and devastating impact of cancer communicated by survivors evoked a sense of anxiety among the interviewed adolescents. Indeed, for all participants a major motivation for sun protection was preventing skin cancer. Although sun exposure is also associated with photo-aging of skin, this was not mentioned in the interviews [18], unlike in other studies where concerns about self-image were reported [19]. Other important considerations include those related to the gender-based differences in attitudes towards sun-protective behaviours observed here, for future studies that test how effective the developed *Sun Safe* app is for modifying the behaviours of males compared to females.

## Data Availability

The datasets used and/or analysed during the current study are available from the corresponding author on reasonable request.

## Abbreviations

PE: Physical education
SPF: Sun protection factor
UV: Ultraviolet
WA: Western Australia
